# A continuous evolution system for contracting the host range of bacteriophage T7

**DOI:** 10.1038/s41598-019-57221-0

**Published:** 2020-01-15

**Authors:** Tzvi Holtzman, Rea Globus, Shahar Molshanski-Mor, Adam Ben-Shem, Ido Yosef, Udi Qimron

**Affiliations:** 1 Department of Clinical Microbiology and Immunology, Sackler School of Medicine, Tel Aviv University, Tel Aviv, 69978 Israel; 2 Department of Integrated Structural Biology, Equipe labellisée Ligue Contre le Cancer, Institut de Génétique et de Biologie Moléculaire et Cellulaire, Illkirch, 67404 France; 3 Present Address: Department of Biotechnology, Israel Institute for Biological Research, Ness Ziona, 74100 Israel

**Keywords:** Microbiology, Bacteriology

## Abstract

Bacteriophage T7 is an intracellular parasite that recognizes its host via its tail and tail fiber proteins, known as receptor-binding proteins (RBPs). The RBPs attach to specific lipopolysaccharide (LPS) features on the host. Various studies have shown expansion of the phage’s host range via mutations in the genes encoding the RBPs, whereas only a few have shown contraction of its host range. Furthermore, most experimental systems have not monitored the alteration of host range in the presence of several hosts simultaneously. Here we show that T7 phage grown in the presence of five restrictive strains and one permissive host, each with a different LPS form, gradually avoids recognition of the restrictive strains. Remarkably, avoidance of the restrictive strains was repeated in different experiments using six different permissive hosts. The evolved phages carried mutations that changed their specificity, as determined by sequencing of the genes encoding the RBPs. This system demonstrates a major role for RBPs in narrowing the range of futile infections. The system can be harnessed for host-range contraction in applications such as detection or elimination of a specific bacterial serotype by bacteriophages.

## Introduction

Bacteriophages (phages) are ubiquitous biological entities that are found in habitats occupied by bacteria. As such, they influence the ecosystem of their habitat. Phages and bacteria exert mutual selection pressures in a never-ending molecular arms race. Therefore, phages must evolve and adapt to changing conditions. One of the ways in which phages can adapt is through changes in their host range.

A bacteriophage’s host range is defined as the span of hosts that it is capable of infecting^1^. This range is dependent on host factors (e.g., defense mechanisms such as CRISPR-Cas^2^ and restriction–modification systems^3^, presence of phage receptors^4^), environmental factors (e.g., temperature and pH^5,6^), and features encoded by the phage (e.g., receptor-binding proteins (RBPs)^7^). Specialist bacteriophages commonly display a narrow host range, i.e., they infect a limited number of bacterial strains of the same species. In contrast, generalist bacteriophages inherently display a broad host range^8,9^. It has been shown that the host range can be artificially expanded^7^. Similarly, host-range contraction is possible; it was shown, for example, that bacteriophage lambda can evolve to improve its binding to a specific receptor while losing the ability to bind another previously recognized receptor^10^.

From an evolutionary point of view, the host range of a phage can be paralleled to animals that choose one food source over another, or specialize in foraging and digesting a specific food source. Bull and colleagues^11^ built a mathematical model of phage–bacterium interactions based on the optimal foraging theory. They used phage T7 to validate their mathematical predictions by demonstrating its ability to discriminate between two bacterial strains. It could evolve to infect one strain of *Escherichia coli* while avoiding another strain that differed only by its surface molecules. They found that a single phage gene, 17, was responsible for the discrimination between the hosts.

**Figure 1:**
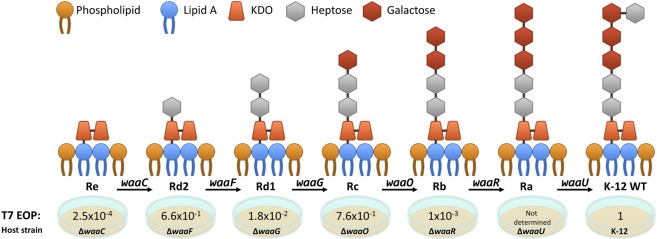
A simplified diagram of the LPS forms displayed on the outer membrane of mutants used in this study. The indicated genes encode enzymes required for the respective LPS biosynthesis. The indicated EOP of phage T7 on the different mutant hosts compared to K-12 is an average of six independent experiments.

Gene 17 encodes the tail fiber proteins that are essential for absorption because they recognize the outer membrane lipopolysaccharide (LPS) of *E. coli*. LPS molecules are abundant on the bacteria’s outer membrane; they are composed of lipid A, which is embedded in the outer membrane, and an appended core region containing keto-deoxy-d-manno-8-octanoic acid, heptose, galactose, and glucose. The biosynthetic pathway of the core region is partly catalyzed by enzymes encoded by the genes *waaC*, *waaF*, *waaG*, *waaO*, *waaR*, and *waaU. E. coli* hosts lacking these genes can be constructed, with the exception of *waaU* which is presumed to be an essential gene^12,13^. Deletion of any one of the other genes results in mutant forms of the LPS presented on the outer membrane of *E. coli*, as depicted in Fig. 1.

We wanted to explore whether bacteriophage T7 can adapt and evolve to distinguish between altered forms of its natural LPS receptor. Specifically, we aimed to evolve T7 specialists that can recognize hosts with an altered LPS form, while avoiding hosts with other LPS forms and even with the wild-type (WT) form. Unlike Bull and colleagues^11^ who propagated T7 phage on a mixture of two hosts at a time, we evolved T7 in a mixture of up to six hosts—each with a different LPS form. Furthermore, we used a continuous evolution system that enabled us to propagate ~100 phage generations in as little as 95 h. In any given experiment, only one host in the mixture was permissive for the propagation of T7, while the other strains were restrictive due to deletion of *trxA*, an essential gene for T7 phage replication. *trxA* encodes thioredoxin that serves as a subunit of the phage DNA polymerase^14^. While its absence consequently halts phage replication and propagation, it does not affect adsorption and DNA injection. This setup directed the evolution of phage T7 toward recognizing a specific LPS form while avoiding the other forms. Using this method, we evolved six different specialist T7 phages, having mutations in tail genes 11 and 12, and in tail fiber gene 17. The evolved phages all showed decreased recognition of LPS forms other than the one they evolved to recognize. These experiments suggest that a major role of RBPs is to avoid restrictive strains, thereby preventing futile injection of the phage DNA.

## Results

### Bacteriophage T7 Evolves to Avoid Its Natural Receptor

We hypothesized that if we use T7 phage to infect two types of host: (1) a strain displaying a poorly recognized LPS but permissive for phage propagation and (2) a strain displaying the natural LPS receptor but restrictive for phage propagation, the phage will evolve to avoid its natural receptor and concomitantly increase its recognition of the permissive host. To test our hypothesis, we used two *E. coli* K-12 BW25113 strains (henceforth K-12) lacking either *trxA* (K-12Δ*trxA*) or *waaC* (K-12Δ*waaC*). *E. coli* lacking *waaC* displays only a partial LPS host receptor, resulting in an efficiency of plating (EOP) of only 2.5 × 10^−4^ of that of the K-12 strain (Fig. 1). In contrast, *E. coli* K-12 hosts lacking *trxA* enable efficient recognition and subsequent absorption, but the infection is abortive because the phage cannot propagate inside the host.

**Figure 2:**
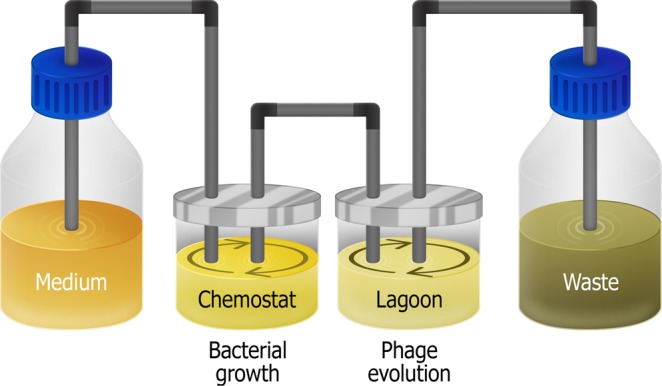
Illustration of the system used for the experiments. LB medium is flown by a pump to a chemostat. Bacteria are flown by a pump into a lagoon in a rate that maintains logarithmic growth. T7 bacteriophage are inoculated initially in the lagoon where they continuously infect bacteria. Both the chemostat and lagoon are diluted at a constant rate by a pump that flows media into a waste container.

*E. coli* strains Δ*trxA* and Δ*waaC* were grown in separate flasks to a similar optical density at 600 nm (OD_600_) of ∼0.2, then mixed in equal volumes and transferred to a continuous evolution system based on a simple chemostat. In this system, *E. coli* are continuously grown in the chemostat with constant influx of fresh Luria-Bertani (LB) medium. They then flow to a second vessel called ‘lagoon’ (Fig. 2). The flow rate from the chemostat is fixed so that the *E. coli* are in the logarithmic growth phase when they enter the lagoon. The stay time of the bacteria in the lagoon is less than the time needed for *E. coli* to replicate, to avoid the accumulation of bacterial mutants. The lagoon contains bacteriophage T7, resulting in phage propagation. The replication time of the phage is shorter than the dilution rate of the lagoon, to avoid phage washout and thus enable selection of the fittest phages.

**Figure 3:**
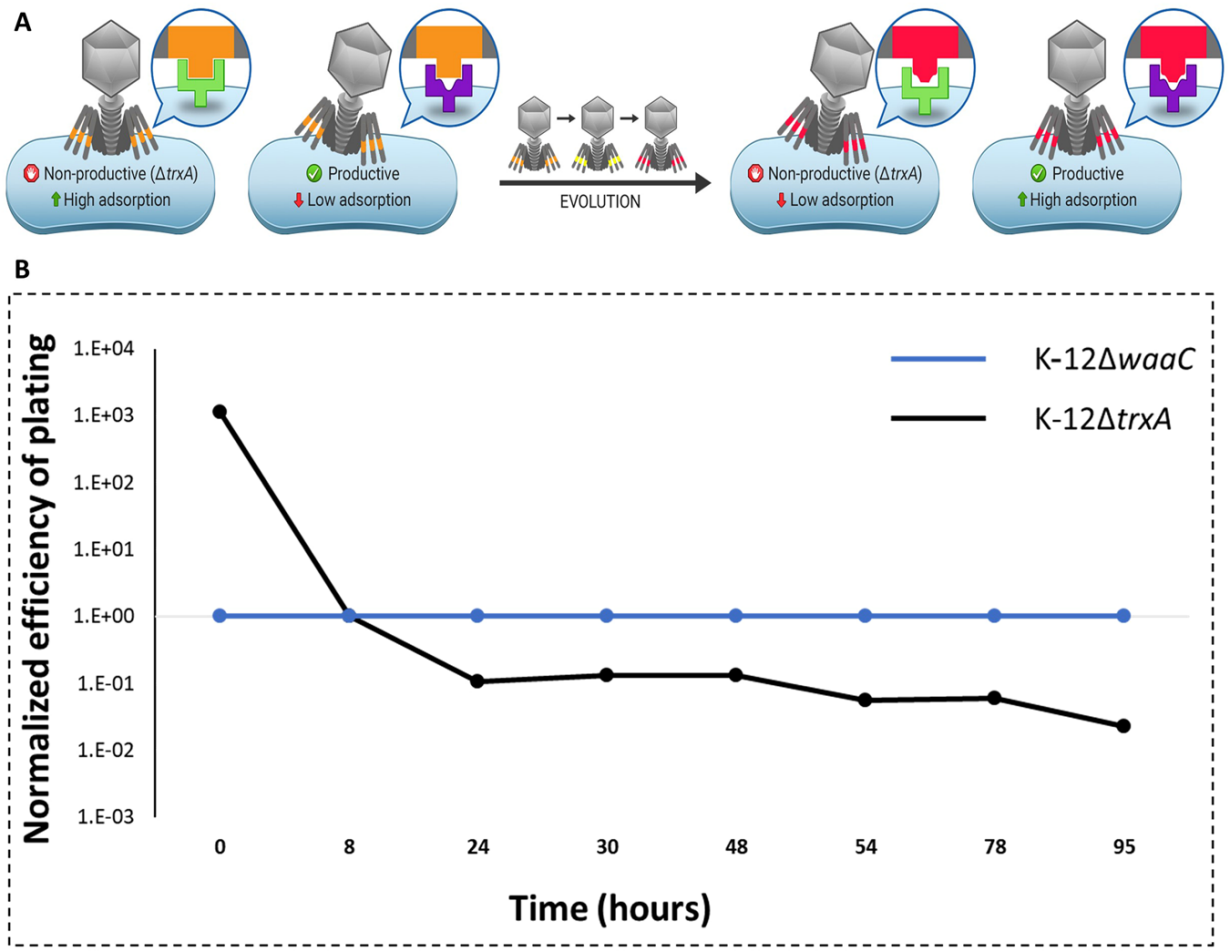
Evolution of phage T7 in the presence of two strains. (**A**) schematic illustration of the evolution. At the starting point of the experiment, most phages recognize the non-productive strain efficiently, while recognition of the permissive host is minimal. After evolving, most of the phage recognize the permissive strain most efficiently, while avoiding the non-productive host. (**B**) T7 phage was propagated in a continuous evolution experiment in the presence of K-12ΔwaaC permissive host and K-12ΔtrxA restrictive strain. Samples were collected at the indicated time points (dots) and the T7 plaque formation was determined on permissive isogenic strains encoding trxA. EOPs were normalized to the EOP of the permissive host.

The system was run for 95 h, allowing replication for ~100 generations of phage. Samples were taken from the lagoon upon addition of the phage (T0) and every 6–24 h thereafter. Phages were extracted from each sample and their EOP was tested on (1) the strain on which they were selected, *i.e*., the permissive host (in this case K-12Δ*waaC*) and (2) a host displaying similar LPS receptors as the restrictive strain, but nonrestrictive as it encodes *trxA*. The latter strain contained a deletion of a control gene, *ydhQ*, which for simplicity will henceforth be omitted from the strains’ description in the main text (but not in Methods) as it does not change the LPS or any known parameter of T7 phage infection^13^. Figure 3 shows the changes in EOP over the course of 95 h. The permissive host served as the reference strain, and hence its value was fixed as 1. The EOP of the restrictive strain was calculated with respect to the permissive host. We used EOP measurements as they are technically easier to carry out in large scale compared to alternatives such as adsorption measurements. The EOP provides a relative number of plaque-forming units compared to a control, and in the absence of propagation barriers it also reflects on the actual adsorption of the phage.

This calculation expresses the difference between the EOP on the two host strains and how it changes over time as the phage evolves. Indeed, as hypothesized, the evolved phage reduced its recognition of the WT LPS form as determined by its EOP on the K-12 strain. While at T0 the phage infected K-12 hosts ∼3 orders of magnitude more efficiently than K-12Δ*waaC* hosts, at T95 the evolved phage changed its differentiation of the two hosts by ∼5 orders of magnitude overall (Fig. 3). It recognized the permissive host by ∼2 orders of magnitude more efficiently than the WT phage, whereas it reduced its recognition of the K-12 strain by ~3 orders of magnitude compared to the WT phage (Dataset S1).

### Bacteriophage T7 Evolves to Avoid Recognition of Restrictive Strains

**Figure 4:**
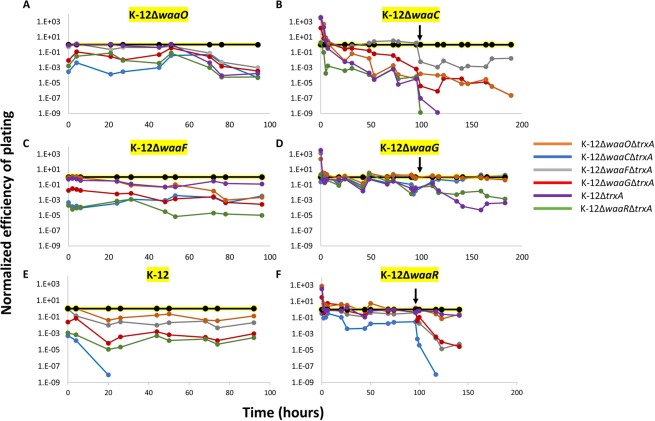
Evolution of phage T7 in the presence of six strains. T7 phage was propagated in a continuous evolution experiment in the presence of one permissive host (solid black line highlighted in yellow in each panel) and the other five restrictive strains (normal lines). Samples were collected at the indicated time points (dots) and the T7 plaque formation was determined on permissive isogenic strains encoding trxA. EOPs were normalized to the EOP of the permissive host. An arrow in panels B, D, and F indicates time of treatment with the EMS mutagen.

We thus demonstrated that the continuous evolution system can select phages that avoid a restrictive strain displaying the natural receptor while recognizing permissive hosts having a poorly recognized receptor. We hypothesized that we could further evolve bacteriophage T7 to avoid multiple LPS forms simultaneously. To this end, we designed subsequent experiments that were similar to the first experiment. However, each experiment had six strains in total: five restrictive strains, and one permissive host. Each of these six strains displayed a different form of LPS, including the WT form. For example, to positively select for phages that specialize in infecting the Rc LPS form, which was found on a host lacking *waaO*, we used a permissive host lacking *waaO* (K-12Δ*waaO*) and mixed it in the lagoon with equal volumes of additional *E. coli* hosts presenting all other LPS forms: the WT lacking trxA (K-12Δ*trxA*), and another four hosts lacking *trxA* and *waaC, waaF, waaG*, or *waaR* (K-12Δ*trxA*Δ*waaC*, K-12Δ*trxA*Δ*waaF*, K-12Δ*trxA*Δ*waaG*, and K-12Δ*trxA*Δ*waaR*, respectively). The system was run for 95 h with samples taken every 4–19 h. The EOPs of the phage samples were determined on all six strains, all encoding *trxA* to enable plaque formation (K-12Δ*waaO*, K-12Δ*waaC*, K-12Δ*waaF*, K-12Δ*waaG*, K-12Δ*waaR*, K-12). Remarkably, after 94 h, the evolved phage infected all of the restrictive strains at least 1000 times less efficiently than the permissive host (Fig. 4A). As expected, the evolved phage exhibited substitutions in gene product (Gp) 17: R200C, G479R, and R542S; Gp12: P694H; Gp11: S15P, all of which were likely responsible for the specialization (Table 1).

**Table 1:** Amino acid substitutions accumulated in RBPs following growth in the presence of the indicated host and five restrictive strains.

Protein	Substitution	Permissive host					
		Δ*waaO*	Δ*waaC*	Δ*waaF*	Δ*waaG*	Δ*waaR*	K-12
**Gp11**	S15P	**X**					
	A40V		**X**				
	V79A			**X**			
**Gp12**	K12R		**X**				
	Y24H				**X**		
	E306K						**X**
	N338G		**X**				
	F467S					**X**	
	D560G				**X**		
	P694S					**X**	
	P694H	**X**			**X**		**X**
	E696G			**X**			
	I779F		**X**				
**Gp17**	T117I					**X**	
	N138S			**X**			
	N158Y						**X**
	R200C	**X**					
	R207Q				**X**		
	K211E		**X**				
	K214T					**X**	
	Y269F				**X**		
	G479R	**X**			**X**		
	G480E		**X**				
	G480V					**X**	
	T485P				**X**		
	C499F					**X**	
	D520E					**X**	**X**
	G521R		**X**				
	G521S			**X**	**X**		
	G534R					**X**	
	D540N					**X**	
	R542S	**X**					

**Figure 5:**
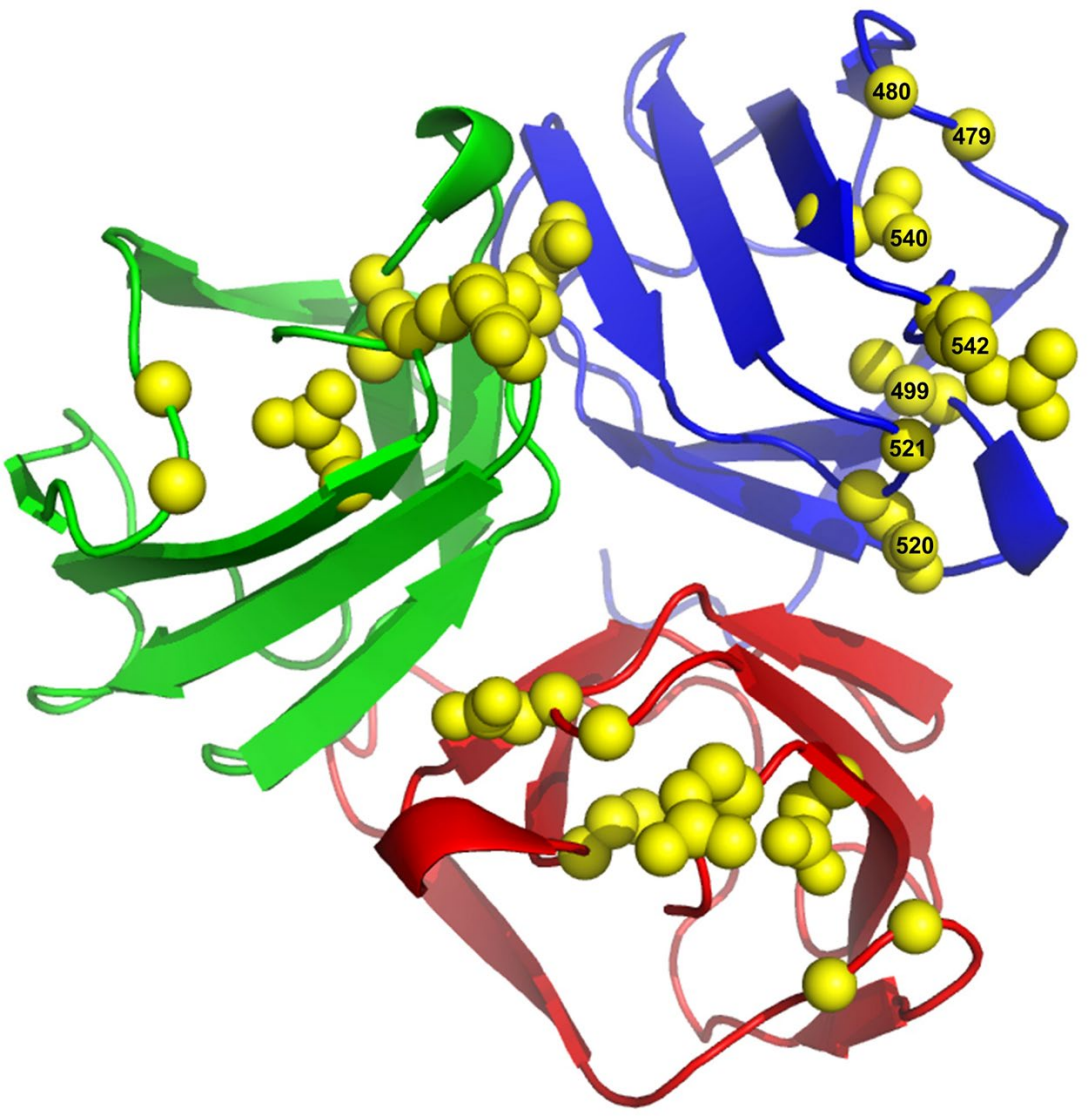
Top view of the crystallographic structure of the carboxy-terminal domain of bacteriophage T7 tail fiber Gp17 containing residues 371–553 (PDB: 4A0T^16^). Side chains of residues G479, G480, C499, D520, G521, D540, and R542 are depicted in space-filled representation (labeled only in one of the three monomers). These substitutions cluster at the top of the tip domain which likely forms the LPS recognition surface.

An additional five similar experiments were carried out by changing the permissive strain to display a different LPS form (Fig. 4B–F). Genes 11, 12, and 17 in the evolved phages were sequenced in all experiments, and the amino acid substitutions are summarized in Table 1. Many amino acid substitutions in Gp17 (residues 479, 480, 499, 520, 521, 540, 542) cluster to the very top of the tip domain, which forms a surface that likely directly interacts with and recognizes the LPS of *E. coli* (Fig. 5). These experiments showed that the WT T7 phage can evolve to better recognize a specific LPS form and simultaneously avoid other LPS forms.

Unexpectedly, when we positively selected for a phage recognizing the LPS form of K-12Δ*waaC*, we found that after 95 h, the evolved specialist phage did not avoid the host K-12Δ*waaF* (Fig. 4B). The LPS of K-12Δ*waaF* contains one heptose group, which is lacking in K-12Δ*waaC* (Fig. 1). We wanted to obtain a specialist phage that differentiates between these two hosts, in addition to the other hosts, which it discriminated against by at least 1000-fold. To this end, we facilitated mutations by treating the phages that evolved for 95 h with the mutagenic chemical ethyl methanesulfonate (EMS). Next, the treated phages were reintroduced to the hosts in the lagoon of the continuous evolution system for a second time, for an additional 94 h. During that time, the EOP on all strains was measured. Remarkably, the EOP for K-12Δ*waaF* was now reduced by ∼2 orders of magnitude, demonstrating that the facilitated evolution enabled stricter avoidance of futile hosts. Moreover, we found that phages sampled after 4 h (99 h total in both rounds of the system) completely lost recognition of the restrictive K-12Δ*waaR*, and after 21 h (116 h total), the phages lost recognition of the restrictive K-12. Similarly, when the permissive host was K-12Δ*waaG*, we found that the evolved phage did not adequately avoid the restrictive strains K-12Δ*waaO* and K-12Δ*waaF* after 95 h (Fig. 4D), and when the permissive host was K-12Δ*waaR*, the evolved phage did not adequately avoid the restrictive strains K-12Δ*waaO* and K-12Δ*waaG* after 95 h (Fig. 4F). EMS treatment in these cases also reduced recognition of the restrictive strains by accumulating mutations in genes 11, 12, and 17 (Fig. 4D,F; Table 1). These experiments demonstrated that with facilitated mutation rates or presumably, with longer evolution time, the phages evolve to further de-recognize and discriminate against restrictive strains.

## Discussion

We showed simultaneous contraction of phage T7’s host range to exclude five different hosts in six different experimental systems. In all cases, we observed that the phages evolved to discriminate against restrictive strains. However, the evolved phages did not always improve their recognition of the permissive host. These findings highlight the notion that a significant role for RBPs is in avoiding restrictive strains, in addition to recognizing permissive hosts. This concept can be envisioned by imagining that the first common ancestor of all current phages developed a simple mechanism to recognize bacterial membranes, which did not require a complex protein structure. Presumably, merely displaying a hydrophobic surface would suffice for such recognition. However, due to the non-selective recognition, some of the progeny of this ancestor infected bacteria that did not support their growth, thus becoming extinct. We speculate that a RBP gradually evolved to avoid recognition of these nonproductive bacterial strains, thus providing a competitive advantage over those phages with the simpler recognition elements. The common perception that RBPs evolved to positively signal the phage as to which host to infect probably stems from observations on host range-expansion observed in the evolution of certain phages^15^. In our system, nevertheless, the RBP is more of a negative signal, indicating primarily where the phage should not attach, rather than being a positive signal for host recognition.

Further support for RBPs evolving to restrict recognition of futile hosts stems from our experiments using K-12 as the permissive host. Arguably, the T7 phage RBPs are fully adapted to recognizing K-12 LPS. When K-12 was used as the permissive host, we observed loss of the T7 phage’s ability to recognize other LPS forms. For example, in less than 20 h of continuous evolution, it completely avoided K-12Δ*waaC*, and markedly avoided K-12Δ*waaG*. We thus demonstrated that the RBP has the potential to avoid other forms of LPS, but does so only if those forms are displayed by restrictive strains. In this case too, the RBP did not evolve to better recognize the K-12 LPS, but only to avoid other hosts. These results reiterate the major role of the RBPs in avoiding restrictive strains.

The latter experiment raises the question of why T7 phage reserves residual recognition of these mutant hosts in the first place, when it can evolve to avoid them efficiently? The likely answer is that these hosts are permissive in nature, and the phage retains the option to infect them, should the host display such mutant receptors. If the phage evolved to avoid residual recognition of those receptors, the bacterial host would evolve to display only those receptors and the phage population would become extinct. We therefore postulate that, in nature, the RBPs of most phages also recognize the derivatives of the major receptor that may be displayed by mutated hosts, but avoid any other receptors that are displayed by restrictive strains. In this respect, it is noteworthy that host restriction in nature can occur in various ways such as restriction-modification systems, CRISPR-Cas systems, and abortive infection systems.

Many of the substitutions we identified clustered at the tip of Gp17. Previously identified substitutions in Gp17 involved in host avoidance were also located on this surface^11^. Intriguingly, the other large concentration of substitutions occurs at the phage-proximal rod (residues 150–260), that is located 10–15 nm away from the tip domain^16,17^. Possibly the proximal rod can also interact with bacterial receptors at some stage of viral infection or alternatively, mutations in the rod could modify the orientation of the tip domain with respect to the bacterial surface, indirectly regulating interactions with LPS. Several substitutions appear also in Gp11 and Gp12 suggesting that the nozzle of the viral DNA injection machinery can also adapt to varying LPS compositions. It is also noteworthy that no substitutions were found in the pyramid domain between the phage-proximal rod and the tip, suggesting that this domain is not involved in avoidance of undesired LPS receptors.

In addition to the theoretical implications of this study, we demonstrate how a continuous evolution system can be used for host-range contraction of bacteriophages. This system may be useful for other research areas that utilize phages. For example, a narrow host range is desirable in bacteriophage therapy. A phage that is limited to a specific species, and even a strain within that species, is sometimes sought because it is likely to minimally interact with other bacteria, and it will therefore not affect the host’s microbiome. It might also help produce phages that are more likely to overcome regulatory demands due to their specific host range. Furthermore, phages are used to identify specific bacteria^18^. Contracting the phage host range to desired bacteria may provide specific identification of certain serotypes. Thus, this study provides both conceptual and translational implications for phage biology and phage applications.

## Materials and Methods

### Reagents

LB medium (10 g/L tryptone, 5 g/L yeast extract, and 5 g/L NaCl) and agar were from Acumedia. Antibiotics were from Calbiochem. EMS was from Sigma-Aldrich. Kappa® High-Fidelity Polymerase was from Kappa Biosystems.

### Strains

The bacterial strains used in this study are listed in Table S1. Single knock-out mutants, BW25113Δ*waaC*, BW25113Δ*waaF*, BW25113Δ*waaG*, BW25113Δ*waaR*, BW25113Δ*waaO*, BW25113Δ*trxA*, and BW25113Δ*ydhQ* were acquired from the Keio collection^12^. IYB5709 is a strain lacking *trxA* from the Keio collection^12^ that was flipped for its resistance cassette using pCP20 as previously described^19^. It was then used as an ancestor strain in homologous recombination to produce double mutants as described below. The WT bacteriophage T7 used in this study has a Gp12 S694P substitution compared to the published sequence (GenBank accession no. CAA24430.1), but is otherwise similar to GenBank AY264774. Phage-generation time during continuous evolution was calculated as previously described^20^.

### Homologous Recombination

Homologous recombination to produce double mutants was performed as previously described^21^. Briefly, an overnight culture of IYB5709 harboring pSIM6 was diluted 70-fold in 35 mL LB medium supplemented with appropriate antibiotics and grown at 32 °C with shaking to an OD_600_ of ∼0.5. The culture was heat-induced at 42 °C for 15 min to express the lambda Red proteins. Then the culture was placed on ice, and pelleted at 3,600 *g* at 0 °C for 10 min. The pellet was washed twice in ice-cold double-distilled H_2_O (ddH_2_O) and then resuspended in 200 µL ice-cold ddH_2_O and kept on ice until electroporation with 500 ng of a gel-purified PCR product. The different PCR products were amplified from the Keio collection strains listed in Table S1. Each PCR product encoded a kanamycin resistance gene with a minimum of 50-bp flanking ends homologous to the target gene. Primers used to produce the different cassettes are listed in Table S2. A 50-µL aliquot of electrocompetent cells was used for each electroporation in a 0.1-cm cuvette. After electroporation, the bacteria were recovered in 1 mL LB medium for 2 h at 32 °C in a shaking water bath and inoculated on selection plates supplemented with 25 µg/mL kanamycin. Homologous recombination into the resulting strains BW25113Δ*trxA*, BW25113Δ*trxA*Δ*waaF*, BW25113Δ*trxA*Δ*waaO*, BW25113Δ*trxA*Δ*waaR*, BW25113Δ*trxA*Δ*waaG*, and BW25113Δ*trxA*Δ*ydhQ*, was confirmed by PCR using the primers IY21F and 21 R to ensure the presence of the *Kan*^*R*^ cassette. In addition, to ensure that the insert entered in the desired location, another PCR was carried out using IY21F and the respective forward or reverse primer that was used to produce the PCR cassette (Table S2).

### Plaque Assay and Lysate Preparation

Plaque assay for quantification of the number of infectious phage particles was performed as previously described^22^. Briefly, chloroform was added to a 5-mL culture of exponentially growing cells mixed with T7 phage extracted from the lagoon of the continuous evolution system at different time points. The lysate was cleared by centrifugation. Then, it was serially diluted and spotted on LB-agar plates with a layer of soft agar containing the appropriate host cells. Plaques were counted and normalized to values obtained by plating on the permissive host.

### EMS Treatment of Phages

Overnight cultures of *E. coli* BW25113Δ*ydhQ* were diluted 1:50 in 5 mL of LB medium supplemented with 25 ug/ml of Kanamycin. The culture was aerated at 37 °C for several hours. Upon reaching mid log-phase, cells were infected with the evolved T7 extracted from the end of each continuous evolution experiment as described above. EMS (Sigma-Aldrich) was then added at 1% to the culture infected with the evolved phage and aerated at 37 °C until lysis appeared. Then, phages were precipitated with PEG 8000 (Promega) and washed twice with 5 mL PBS as previously described^23^.

### Continuous Evolution

A schematic of the system can be found in Fig. 2. The system was composed of two connected 500 mL vessels each contained 125 mL culture, stirred continuously. The first vessel, “chemostat”, was used for *E. coli* growth, and the second vessel, “lagoon”, for viral evolution. Before entering the system, each host strain was grown separately with shaking at 37 °C in 125 mL flask containing 25 mL LB with appropriate antibiotics until reaching ∼0.5 OD_600_. Then, all hosts were mixed in equal parts to total volume of 125 mL and were introduced aseptically into the chemostat. Fresh LB media was added continuously to the chemostat with a flow rate of 2 volumes per hour (2 V/h) until reaching a steady stare of ∼1.0 OD_600_. Then, the chemostat culture was flown into the lagoon with a flow rate of 2 V/h, which was also the flow rate used for fluid removal from the lagoon to the waste container. At T0, T7 bacteriophage was aseptically injected to the lagoon with a MOI of 1. Samples of 15 mL were taken aseptically from the chemostat and from the lagoon via sampling ports, from which, 5 mL were used to prepare a lysate as described above. In order to maintain bacterial strain ratio in the chemostat, 300 mL of freshly prepared bacterial mix with ~1 OD_600_ was aseptically added to the chemostat culture every 24 h. At that time, media supplement to the chemostat vessel was stopped until reaching the initial volume of 125 mL.

## Supplementary information

Supplementary Information.

Supplementary Dataset 1.
