# Supplementary material for: A continuous evolution system for contracting the host range of bacteriophage T7
Source: Sci Rep. 2020 Jan 15;10:307. doi: 10.1038/s41598-019-57221-0 (PMC6962156; doi:10.1038/s41598-019-57221-0)
Supplement: Supplementary file 1 — Supplementary Information. [file 41598_2019_57221_MOESM1_ESM.docx]

Supplementary Information for

**A continuous evolution system for contracting the host range of bacteriophage T7**

**Tzvi Holtzman^1, #, §^, Rea Globus^1, #^, Shahar Molshanski-Mor^1^, Adam Ben-Shem^2^, Ido Yosef^1^, and Udi Qimron^1, *^**

Udi Qimron

Email: [ehudq@post.tau.ac.il](mailto:ehudq@post.tau.ac.il)

Table S1. Bacterial strains used in this study.

| **Strain name** | **Description** | **Source** |
| --- | --- | --- |
| JW5856 | *Escherichia coli* BW25113 Δ*trxA::KanR* | Previous work^1^ |
| JW3596 | *Escherichia coli* BW25113 Δ*waaC::KanR* | Previous work^1^ |
| JW3595 | *Escherichia coli* BW25113 Δ*waaF::KanR* | Previous work^1^ |
| JW3602 | *Escherichia coli* BW25113 Δ*waaO::KanR* | Previous work^1^ |
| JW5856 | *Escherichia coli* BW25113 Δ*waaR::KanR* | Previous work^1^ |
| JW3606 | *Escherichia coli* BW25113 Δ*waaG::KanR* | Previous work^1^ |
| JW1656 | *Escherichia coli* BW25113 Δ*ydhQ::KanR* | Previous work^1^ |
| IYB5709 | *Escherichia coli* BW25113 Δ*trxA* | This study |
| IYB5758 | *Escherichia coli* BW25113 Δ*trxA*Δ*WaaC::Kan^R^* | Previous work^2^ |
| TH4 | *Escherichia coli* BW25113 Δ*trxA*Δ*waaF::Kan^R^* | This study |
| TH5 | *Escherichia coli* BW25113 Δ*trxA*Δ*waaO::Kan^R^* | This study |
| TH6 | *Escherichia coli* BW25113 Δ*trxA*Δ*waaR::Kan^R^* | This study |
| TH7 | *Escherichia coli* BW25113 Δ*trxA*Δ*waaG::Kan^R^* | This study |
| TH8 | *Escherichia coli* BW25113 Δ*trxA*Δ*ydhQ::Kan^R^* | This study |

Table S2. Oligonucleotides used in this study.

| **Oligonucleotides** | **Sequence 5'→3'** | **Note** |
| --- | --- | --- |
| IY139F | TGCTGCCACCGCTGAGCAAT | Gene 11 + Gene 12 amplification prior sequencing |
| IY407R | TGTCATGGTGAGCCGGAGTG | Gene 11 + Gene 12 amplification prior sequencing |
| IY139F | TGCTGCCACCGCTGAGCAAT | Gene 11 + Gene 12 sequencing |
| 142F | AAGCACACGGTCACACTGCT | Gene 11 + Gene 12 sequencing |
| 143F | CAATAAACCGGTAAACCAGC | Gene 11 + Gene 12 sequencing |
| 144F | AAGCGGCTATTTAACGACCC | Gene 11 + Gene 12 sequencing |
| 146F | TGCTGCGAAATTTGAACGCC | Gene 11 + Gene 12 sequencing |
| 147F | CCGCACTCGAGTCTGGTAAA | Gene 11 + Gene 12 sequencing |
| IY294F | TACAGGGAGAACCCTATCGT | Gene 11 + Gene 12 sequencing |
| IY407R | TGTCATGGTGAGCCGGAGTG | Gene 11 + Gene 12 sequencing |
| 167F | ATGAATGCTGCTGGCGTGGT | Gene 17 amplification prior sequencing |
| IY172Ra | GTCCATCCGTGGACTACACG | Gene 17 amplification prior sequencing |
| 167F | ATGAATGCTGCTGGCGTGGT | Gene 17 sequencing |
| IY398F | TAAGGTCGACAAGAAGGAGATATACATATGGCTAACGTAATTAAAACCG | Gene 17 sequencing |
| 168F | CCATGGGACCGTATGTTTCCGA | Gene 17 sequencing |
| 170F | ACTCGCCTATGTATAAACTTTGTGTGAAATTGTTATCCGCTC | Gene 17 sequencing |
| 171F | ACGCTCATATGGCTCGTCTTCACCTCGAGAAATC | Gene 17 sequencing |
| IY172Ra | GTCCATCCGTGGACTACACG | Gene 17 sequencing |
| TH01F | GATTGCAGCCTGTAAAGCC | *Kan^R^* cassette amplification for recombineering into *waaC* |
| TH01R | CCAAATATAAATATTTTGGC | *Kan^R^* cassette amplification for recombineering into *waaC* |
| TH02F | AGTGCTTTTTATTGGTGTGG | *Kan^R^* cassette amplification for recombineering into *waaG* |
| IY129R | TTCACGAAAGACCTCGCCGT | *Kan^R^* cassette amplification for recombineering into *waaG* |
| TH03F | TGTGAATCTGTGGTTCCTGG | *Kan^R^* cassette amplification for recombineering into *waaF* |
| TH03R | GCCAGACGCGTCACCAGCGC | *Kan^R^* cassette amplification for recombineering into *waaF* |
| TH04F | CAATGACCCTACTTGAAGCC | *Kan^R^* cassette amplification for recombineering into *waaI* |
| TH04R | GAAGTACATTGCTCTTGACC | *Kan^R^* cassette amplification for recombineering into *waaI* |
| TH05F | TATAAACCCAGTAACCAACG | *Kan^R^* cassette amplification for recombineering into *waaJ* |
| TH05R | CCCTTCATTTCGTACTTTTT | *Kan^R^* cassette amplification for recombineering into *waaJ* |
| TH06F | TGCGATTCATTGAAACTCGG | *Kan^R^* cassette amplification for recombineering into *waaO* |
| TH06R | AATAAACATCCAGGCCAGGC | *Kan^R^* cassette amplification for recombineering into *waaO* |
| MM40F | CAGAGTGGGAAGGTGGCAAGA | *Kan^R^* cassette amplification for recombineering into *ydhQ* |
| IY33R | GTCAGCCATCATTCTGCAAC | *Kan^R^* cassette amplification for recombineering into *ydhQ* |
| IY21F | TTCCTCGTGCTTTACGGTATCG | *Kan^R^* insert verification |
| IY21R | TGTGCCCAGTCATAGCCGAATAGC | *Kan^R^* insert verification |

Dataset S1. Raw data of EOP experiments, as well as the normalized data used to generate the presented figures.

**SI References**

1. T. Baba *et al.*, Construction of *Escherichia coli* K-12 in-frame, single-gene knockout mutants: the Keio collection. *Mol Syst Biol* **2**, 2006.0008 (2006).

2. I. Yosef, M. G. Goren, R. Globus, S. Molshanski-Mor, U. Qimron, Extending the Host Range of Bacteriophage Particles for DNA Transduction. *Mol Cell* **66**, 721-728.e723 (2017).
